# The Abnormality of Topological Asymmetry between Hemispheric Brain White Matter Networks in Alzheimer’s Disease and Mild Cognitive Impairment

**DOI:** 10.3389/fnagi.2017.00261

**Published:** 2017-08-07

**Authors:** Cheng Yang, Suyu Zhong, Xiaolong Zhou, Long Wei, Lijia Wang, Shengdong Nie

**Affiliations:** ^1^Institute of Medical Imaging Engineering, School of Medical Instrument and Food Engineering, University of Shanghai for Science and Technology Shanghai, China; ^2^State Key Laboratory of Cognitive Neuroscience and Learning and IDG/McGovern Institute for Brain Research, Beijing Normal University Beijing, China; ^3^Laiwu Vocational and Technical College Shandong, China

**Keywords:** Alzheimer’s disease, connectome, diffusion tensor imaging, graph theory, hemispheric asymmetry, mild cognitive impairment, lateralization

## Abstract

A large number of morphology-based studies have previously reported a variety of regional abnormalities in hemispheric asymmetry in Alzheimer’s disease (AD). Recently, neuroimaging studies have revealed changes in the topological organization of the structural network in AD. However, little is known about the alterations in topological asymmetries. In the present study, we used diffusion tensor image tractography to construct the hemispheric brain white matter networks of 25 AD patients, 95 mild cognitive impairment (MCI) patients, and 48 normal control (NC) subjects. Graph theoretical approaches were then employed to estimate hemispheric topological properties. Rightward asymmetry in both global and local network efficiencies were observed between the two hemispheres only in AD patients. The brain regions/nodes exhibiting increased rightward asymmetry in both AD and MCI patients were primarily located in the parahippocampal gyrus and cuneus. The observed rightward asymmetry was attributed to changes in the topological properties of the left hemisphere in AD patients. Finally, we found that the abnormal hemispheric asymmetries of brain network properties were significantly correlated with memory performance (Rey’s Auditory Verbal Learning Test). Our findings provide new insights into the lateralized nature of hemispheric disconnectivity and highlight the potential for using hemispheric asymmetry of brain network measures as biomarkers for AD.

## Introduction

Alzheimer’s disease is the most common form of dementia, comprising an estimated 60-80% of all dementia cases, and MCI is considered a prodromal state of AD and possibly other dementias in which cognitive decline is greater than expected at a certain age or education level, but with functional impairment that is insufficient for a diagnosis of dementia (Petersen et al., 1985). Previous neuroimaging studies have demonstrated aberrant hemispheric asymmetry of both brain structure and function in AD and MCI, including cortical thickness (Kim et al., 2012), cortical volume (Müller et al., 2005; Pennanen et al., 2005; Shi et al., 2009; Cherbuin et al., 2010; Derflinger et al., 2011; Dhikav et al., 2016; Li et al., 2016), and cortical surface area covarying across the brain (Thompson et al., 2003, 2007; Long et al., 2013). There is also aberrant hemispheric asymmetry in WM properties (Müller et al., 2005; Damoiseaux et al., 2009; Stricker et al., 2009; Liu et al., 2011; Wessa et al., 2016) and functional connectivity (Wang et al., 2006, 2015). Intriguingly, some of these studies showed that the left hemisphere was significantly more impaired than the right, indicating a faster left hemisphere degeneration in AD (Thompson et al., 2003, 2007; Müller et al., 2005; Damoiseaux et al., 2009; Li et al., 2016; Wessa et al., 2016).

Recently, the term “connectome” was proposed by modeling the human brain as a complex network, and graph theoretical approaches provide powerful tools to uncover human brain connectome in healthy and disease populations (Sporns et al., 2005; Bullmore and Sporns, 2009; Rubinov and Sporns, 2009; Fornito et al., 2013, 2015; Fornito and Bullmore, 2015; Ohno et al., 2016). Diffusion MRI is the only non-invasive neuroimaging technique that can provide exquisite details of WM fiber bundles *in vivo*, and have been extensively applied to study AD and MCI (Douaud et al., 2011; Liu et al., 2011; Bosch et al., 2012; Huang et al., 2012; Voineskos et al., 2012; Amlien and Fjell, 2014). Furthermore, the WM connectome constructed by using diffusion MRI have shown altered topological properties in AD and MCI (Dai and He, 2014; Daianu et al., 2014, 2015; Garcés et al., 2014; Phillips et al., 2014; Prescott et al., 2014; Sun et al., 2014; Zhan et al., 2015; Zhang et al., 2015; Palesi et al., 2016; Wang et al., 2016). Intriguingly, Daianu et al. (2013) found significant differences between the left and right hemispheric in the k-core matrices among NC, AD, and MCI. Specifically, the entire k-core was “lost” in the left hemisphere of AD subjects, supporting lateralized deficits in connectome topologies of AD.

To date, whether and how the asymmetry of hemispheric network topology differs among AD, MCI and normal aging remain largely unknown. Given the difference in hemispheric asymmetries previously observed among AD, MCI and normal aging, we hypothesized the existence of differences in the topological asymmetry of hemispheric networks. To test this hypothesis, diffusion magnetic resonance imaging (MRI) was utilized to construct hemispheric brain WM networks for AD patients, MCI patients, and normal control (NC) subjects. Graph theoretical methods were then applied to quantify multiple topological parameters for the hemispheric networks.

## Materials and Methods

### Participants

All participants in this study were selected from the second stage of the ADNI-II database, available at http://adni.loni.usc.edu/. Written informed consent was obtained, as approved by the Institutional Review Board at each participating center. ADNI is a large multi-site longitudinal study for evaluating biomarkers of AD. The diagnostic categories are based on the online standard criteria^1^. **Table 1** illustrates the demographic information of all subjects, and only subjects who acquired diffusion MRI data using a 3T GE Medical System scanner were included in the present study. The diagnostic inclusion criteria for each group were as follows:

**Table 1:** Demographic information of study participants.

	NC	MCI	AD
No. of subjects	48	95	25
Age range (years)	60–90	57–94	62–92
Age (mean ± SD)	75.0 ± 6.0	74.9 ± 7.4	77.4 ± 8.4
Sex (Male/Female)^∗^	21/27	57/38	19/6
MMSE (mean ± SD)^∗∗^	29.19 ± 1.14	27.23 ± 1.59	22.6 ± 2.02
TOTAL (mean ± SD)^∗∗^	43.69 ± 11.01	32.56 ± 10.28	20.92 ± 5.48
TOT6 (mean ± SD)^∗∗^	8.35 ± 3.91	4.89 ± 3.40	1.08 ± 1.11
RECOG (mean ± SD)^∗∗^	6.90 ± 4.26	3.26 ± 3.43	0.36 ± 0.63


*^∗^Indicates significant difference across groups (*p* < 0.05), as assessed by a chi-square test.*

*^∗∗^Indicates significant difference across groups (*p* < 0.001), as assessed by one-way ANOVA.*

#### Normal Control Subjects

Mini-Mental State Examination scores between 24 and 30 (inclusive), a CDR of 0, and the absence of depression or dementia.

#### MCI Subjects

Mini-Mental State Examination scores between 24 and 30 (inclusive), a CDR of 0.5, with memory complaint and objective memory loss measured by education-adjusted scores on the Wechsler Memory Scale Logical Memory II, absence of significant levels of impairment in other cognitive domains, essentially preserved activities of daily living, and absence of dementia.

#### Alzheimer’s Disease Subjects

Mini-Mental State Examination scores between 20 and 26 (inclusive) and a CDR of 0.5 or 1.0.

### Neuropsychological Testing

To explore the relationship between AI and the memory, Rey’s Auditory Verbal Learning Test (RAVLT) were chosen from the ANDI cognitive battery. Previous studies have proven the effectiveness of RAVLT scores in evaluating memory complaints caused by AD (Balthazar et al., 2010; Reijmer et al., 2013; Moradi et al., 2016). The RAVLT begins with a list of 15 unrelated words (List A) repeated over 5 different trials, then the participant is asked to repeat as many words as possible (Trials 1 to 5). After that, a new list (List B) of 15 new words is read to the participant, who is immediately asked to recall the words. After the List B trial, the examiner asks participant to recall the words from the List A (Trial 6). After 30-min of interpolated testing (timed from the completion of List B recall), the participant is again asked to recall the words from the List A (delayed recall). In this context, three RAVLT scores were used, i.e., TOTAL score [the sum of scores form 5 first trials (Trails 1 to 5)], TOT6 score (the scores form trails 6), and the RECOG score (30-min delayed recall score). Notably, there were significant group differences in TOTAL score (*F* = 45.29, *p* < 0.001), TOT6 score (*F* = 40.74, *p* < 0.001), and RECOG score (*F* = 32.79, *p* < 0.001), as shown in **Table 1**.

### MRI Data Acquisition

Diffusion-weighted imaging and T1-weighted imaging data were acquired for each participant using 3T GE Medical System scanners at a variety of sites. T1-weighted images were collected using spoiled gradient echo (SPGR) sequences with the following parameters: 256 × 256 acquisition matrix; voxel size = 1.2 mm × 1.0 mm × 1.0 mm; inversion time (TI) = 400 ms; TR = 6.98 ms; TE = 2.85 ms; and flip angle = 11°. The diffusion-weighted images consisted of 41 diffusion-weighted volumes, with b = 1,000 s/mm^2^ and 5 b0 volumes with no diffusion sensitivity and were collected with the following parameters: 128 × 128 matrix; voxel size = 2.7 mm × 2.7 mm × 2.7 mm; number of slices = 59; and scan time = 9 min. The values of TR and TE were not exactly the same because the data were acquired from different sites. More imaging details can be found at http://adni.loni.usc.edu/wp-content/uploads/2010/05/ADNI2_GE_3T_22.0_T2.pdf.

### Hemispheric Brain WM Network Construction

The procedures were implemented using a pipeline tool of diffusion MRI called PANDA (Cui et al., 2012). We first preprocessed all diffusion-weighted images, including brain extraction, correction for eddy-current distortion and simple head motion, correction for b-matrix, and computation for diffusion tensor and FA. To study the topological asymmetry of human brain networks, two hemispheric brain WM networks were constructed for each subject. A brain network consists of two basic elements: nodes and edges.

#### Node Definition for the Hemispheric Brain WM Networks

The nodes were determined by the same procedures as previously proposed (Zhong et al., 2017). First, the automated anatomical labeling (AAL) atlas (Tzourio-Mazoyer et al., 2002) provided by the Montreal Neurological Institute (MNI) was binarized to obtain a basic mask. Then, the left and right hemispheres were flipped along the x-axis, and the basic mask and the flipped version were averaged to generate a symmetric mask. The right side of the symmetric mask was parcellated into a 512-ROI set using a random partition procedure (Zalesky et al., 2010): each ROI represented a node. Second, the resultant 512-ROI set was flipped into the left hemisphere to obtain the final template. This flipping ensured one-to-one correspondence of ROIs/nodes between the left and right hemispheric networks, enabling direct comparison between the two hemispheric networks. Finally, the ROI set in the MNI space was transformed into the native diffusion space for each subject as described in a previous study (Gong et al., 2009). Accordingly, each individual FA image was first co-registered to the T1-weighted structural images in the native diffusion space using a linear transformation. The co-registered structural images were then non-linearly normalized to the symmetric ICBM-152 T1 template in MNI space. Finally, the inverse transformations were applied to the parcellation of MNI space, resulting in native-space GM parcellations for each subject.

#### Edge Definition for the Hemispheric Brain WM Networks

The whole-brain fiber tracking was performed in native diffusion space for each subject using the Fiber Assignment by Continuous Tracking (FACT) algorithm (Mori et al., 1999). Fiber tracking continued until the FA value was less than 0.2 or the angle between the current and the previous path segment was higher than 45°. The edge would be built up between a pair of nodes if more than one fiber was connected. In this study, the edge weight was defined as the mean FA values of the connected fibers between two regions. The FA value is an important index for evaluating fiber integrity (Beaulieu, 2002), and several studies have used FA as a marker to study the efficiency of brain connections (Lo et al., 2010; van den Heuvel and Sporns, 2011). Finally, two hemispheric 512 × 512 symmetric weighted matrices were generated for each subject.

### Network Parameters

The topological properties of human brain networks can be analyzed quantitatively using graph theory (Bullmore and Sporns, 2009). In the current study, we calculated the global network efficiency, local network efficiency and nodal efficiency to characterize the topological organization of WM networks at both global and regional levels using GRETNA^2^. A brief description for these network parameters was as follows:

#### Global Network Efficiency

The global efficiency of a network (*E*_glob_) is defined as the average inverse shortest path length (Latora and Marchiori, 2001). For a given network G with N nodes, the *E*_glob_ is computed as follows:

$$E_{glob}^{G}=\frac{1}{N\left(N-1\right)}\underset{i\in G}{\Sigma }\,\;\underset{j\neq i\in G}{\Sigma }\frac{1}{L_{ij}}$$

where *L*_ij_ is the shortest path length between node *i* and node *j. E*_glob_ reflects the information transferring ability of the entire network, and a larger *E*_glob_ correlates with a more powerful efficiency.

#### Local Network Efficiency

The local efficiency of a network (*E*_loc_) is defined as the average of the local efficiencies of all individual nodes (Latora and Marchiori, 2001), which is expressed as follows:

$$E_{loc}^{G}=\frac{1}{N}\underset{i\in G}{\Sigma }E_{glob}^{G_{i}}$$

where *G*_i_ is the sub-graph composed of the nearest neighbors of node *i* and the connections among them. *E*_loc_ corresponds to the average efficiency of information flow within the local environment and reflects the average ability of a network to tolerate faults.

#### Nodal Efficiency

The nodal efficiency for a given node *i* (*E*_nodal_) is defined as the mean of the shortest path length between node *i* and all other nodes in the network (Achard and Bullmore, 2007), that is:

$$E_{nodal}^{G}\left(i\right)=\frac{1}{N-1}\underset{i\neq j\in G}{\Sigma }\frac{1}{L_{ij}}$$

*E*_nodal_ represents the capacity of a node to communicate with the other nodes of a network.

#### Asymmetry Index (AI)

To assess the degree of differences in left and right hemispheric networks, the AI was computed for each of the left-right pairs using the following formula (Iturria-Medina et al., 2011; Sun et al., 2015):

$$AI=\frac{M_{R}-M_{L}}{M_{R}+M_{L}}$$

For network efficiencies, *M_R_* and *M_L_*, respectively stand for the global network or local network efficiency of the right and left hemispheric network. For nodal efficiency, *M*_R_ and *M*_L_, respectively represent the nodal efficiency of corresponding ROIs from the right and left hemispheric network. There are 512 nodes in each hemisphere. Thus, the AI of node efficiency would have 512 values. Notably, a positive value of AI represents a rightward asymmetry, while a negative value of AI indicates a leftward asymmetry.

### Statistical Analysis

We first tested the within-group asymmetries of global network efficiency, local network efficiency, and nodal efficiency for each group (i.e., AD, MCI, and NC). Data were analyzed using GLMs with repeated measures. Specifically, the left and right hemispheres were taken as the repeated variable, while age, sex, and the TR and TE of diffusion-weighted imaging were used as covariates. The ICV of the hemispheres was also included as a covariate in the statistical model, considering the network efficiency-ICV relationship (Yan et al., 2011) and the difference in the hemispheric brain size (Giedd et al., 1999). The hemispheric ICV was computed by summing the volume of WM, GM, and cerebrospinal fluid (CSF) within each hemisphere. The tissue was segmented from the T1-weighted images using SPM8^3^. For network efficiencies, a value of *P* < 0.05 was considered significant. Multiple-comparison correction was performed for nodal efficiency using the FDR method, and *q* < 0.05 was considered significant.

Next, we assessed group differences in the AI of the global network efficiency, local network efficiency, and nodal efficiencies using the GLMs. Notably, the “sex × group” interaction was first evaluated for each AI measure. If the interaction was not significant, the interaction term was excluded in the statistical model. The main group effects were then evaluated after controlling for age, sex, whole-brain ICV, and the difference in hemispheric ICV, TR and TE of diffusion-weighted imaging. For the AI of nodal efficiency (512 in total), the statistical procedure was confined to the nodes/ROIs that showed a significant asymmetry in at least one of the groups from the analysis in the previous step. In addition, the Bonferroni method was used to correct for *post hoc* multiple comparisons (Comparison time is 3). For all parameters, *P* < 0.05 was considered significant.

Besides, to further explore the cause of AI differences, similar group comparisons of global network efficiency, local network efficiency and the mean nodal efficiency were implemented in each hemisphere, with age, sex, and hemispheric ICV, TR, and TE of diffusion-weighted imaging as covariates.

Finally, we examined the relationships between the AI and the RAVLT scores (i.e., TOTAL score, TOT6 score and RECOG score) across all subjects. Specifically, multiple linear regressions were employed taking age, sex, whole-brain ICV, and the difference in hemispheric ICV, TR, and TE of diffusion-weighted imaging as covariates. To limit the number of association calculations for regional properties, only the nodes showing significant group difference in the AI of nodal efficiency were chosen as independent variables. For global network efficiency and local network efficiency, *p* < 0.05 was considered for establishment of a significant relationship. Multiple-comparison correction was performed for nodal efficiency using the FDR method, and *q* < 0.05 was considered significant.

## Results

### Within-Group Asymmetry of Global and Local Network Efficiencies

**FIGURE 1:**
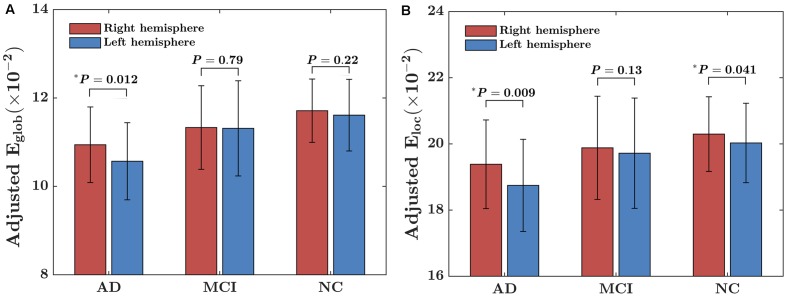
Within-group asymmetry of the global and local network efficiency of the hemispheric networks. **(A)** Global network efficiency (*E*_glob_); **(B)** Local network efficiency (*E*_loc_). For each group, the statistical analysis was conducted after the data were adjusted for age, sex, hemispheric ICV, and TR and TE from diffusion-weighted imaging. ^∗^indicates a significant difference between two hemispheres (*P* < 0.05).

The within-group asymmetries of global and local network efficiencies for each group are illustrated in **Figure 1**. Significant rightward asymmetry in global network efficiency was observed in the AD group (*t* = 2.63, *P* = 0.012) but not in the MCI group (*t* = 0.27, *P* = 0.79) or the NC group (*t* = 1.23, *P* = 0.22). For local network asymmetry, significant rightward (i.e., right > left) asymmetry was observed in both the AD (*t* = 2.72, *P* = 0.009) and NC (*t* = 2.08, *P* = 0.041) group but not in the MCI (*t* = 1.52, *P* = 0.13) group.

### Between-Group Differences in the Asymmetry of Global and Local Network Efficiency

**FIGURE 2:**
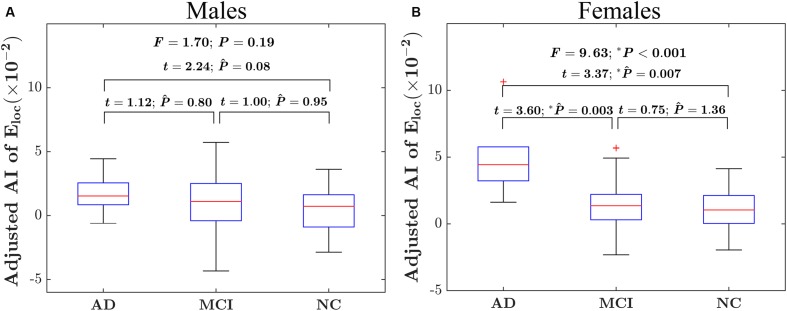
Between-group differences on the AI of the local network efficiency of the hemispheric networks in males and females. **(A)** The box plots of the group differences on the AI of local network efficiency in males. **(B)** The box plots of the group differences on the AI of local network efficiency in females. Before conducting the group comparisons, the data were adjusted for whole-brain ICV, hemispheric ICV difference, age, gender, and TR and TE from diffusion-weighted imaging. $\overset{\;\wedge }{P}$ represents the Bonferroni-corrected *P-*value of *post hoc* analysis.

There were significant “sex × group” interaction effect on the AI of local network efficiency (*F* = 3.19; *P* = 0.04), but no significant interaction effect on the AI of global network efficiency (*F* = 1.08; *P* = 0.34). *Post hoc* analysis showed that group difference was found only in females (*F* = 9.55; *P* < 0.001) but not males (*F* = 1.73; *P* = 0.78), as shown in **Figures 2A,B**. More specifically, for females, there were significantly difference in the asymmetry of local network efficiency between AD group and NC group (*t* = 3.37; *P*_Bonferroni_ = 0.007) and between AD group and MCI group (*t* = 3.60; *P*_Bonferroni_ = 0.003).

**FIGURE 3:**
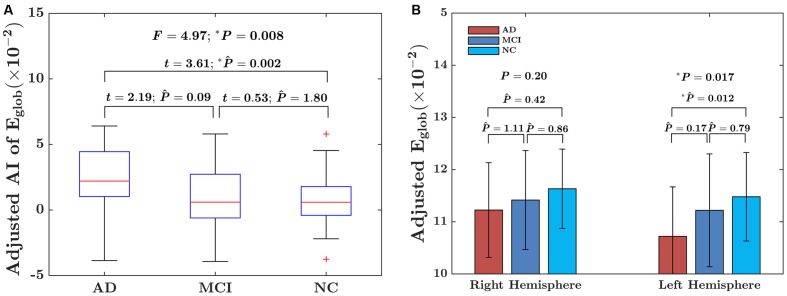
Between-group differences in the AI of the global network efficiency of the hemispheric networks and the absolute network efficiency in the two hemispheres. **(A)** The box plots of the group differences in the AI of global network efficiency. Before conducting the group comparisons, the data were adjusted for whole-brain ICV, hemispheric ICV difference, age, gender, and TR and TE from diffusion-weighted imaging. **(B)** Bar charts depicting group differences in the global network efficiency of each hemisphere. Before conducting the group comparisons for each hemisphere, the hemispheric ICV, age, sex, and TR and TE from diffusion-weighted imaging were adjusted. $\overset{\;\wedge }{P}$ represents the Bonferroni-corrected *P-*value of *post hoc* analysis.

For the global network efficiency, on which there was no significant “sex × group” interaction effect the group effect was explored after excluding the interaction term from the statistical model. There were significant group effects on the AI of global network efficiency (*F* = 4.97; *P* = 0.008) as shown in **Figure 3A**. For the AI of the global network, *post hoc* comparison revealed that there was a significant group difference only between NC and AD group. Compared with NC group, AD group exhibited significantly increased rightward asymmetry in global network efficiency (*t* = 3.61; *P*_Bonferroni_ = 0.002). Regarding the hemispheric topological properties for each hemisphere, there were significant group differences in global network efficiency only in the left hemisphere (*E*_glob_ of left hemisphere: *F* = 4.12 *P* = 0.017; E_glob_ of right hemisphere: *F* = 1.62; *P* = 0.20; **Figure 3B**). There was a trend toward an increase in global network efficiency in both hemispheres from AD to MCI to NC (**Figure 3B**). *Post hoc* comparisons revealed that compared with NC, AD exhibited significantly decreased global network efficiency in the left hemisphere (*E*_glob_: *t* = –2.99; *P*_Bonferroni_ = 0.012), but not in the right hemisphere (*E*_glob_: *t* = –1.49; *P*_Bonferroni_ = 0.42). It is likely that the increased rightward asymmetry in the AD group compared with the NC group is mainly attributed to this observed reduction in global network efficiency of the left hemisphere in AD. There were no significant differences between AD and MCI in either the left hemisphere (*E*_glob_: *t* = –1.93; *P*_Bonferroni_ = 0.17, *E*_loc_: *t* = –1.69; *P*_Bonferroni_ = 0.28) or right hemisphere (E_glob_: *t* = –0.90; *P*_Bonferroni_ = 1.11). There were also no significant differences between MCI and NC in either the left hemisphere (*E*_glob_: *t* = –1.12; *P*_Bonferroni_ = 0.79) or right hemisphere (*E*_glob_: *t* = –1.07; *P*_Bonferroni_ = 0.86).

### Within-Group Asymmetry of Nodal Efficiency

**FIGURE 4:**
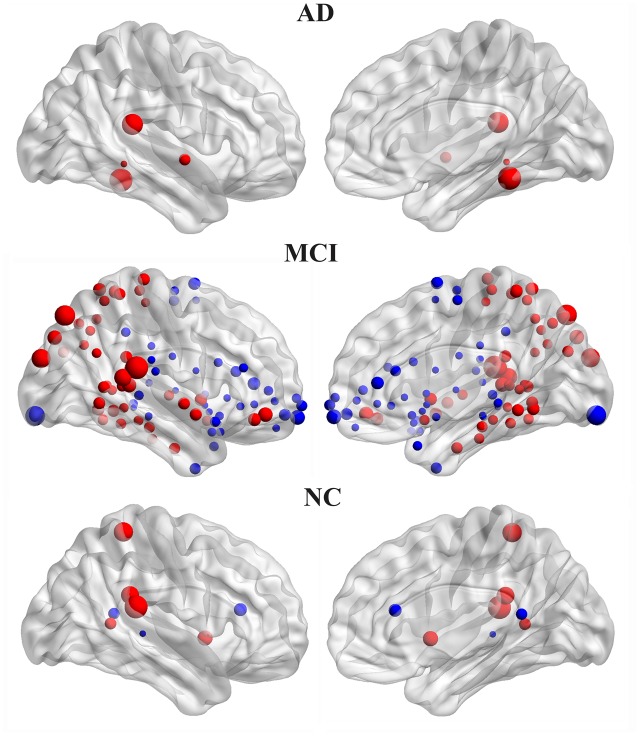
Three-dimensional representations of the significant between-hemisphere differences in nodal efficiency (FDR-corrected *P* < 0.05) in the AD, MCI and NC groups. The nodes shown in red represent the significantly rightward asymmetric nodes, and the nodes in blue represent significantly leftward asymmetric regions. The node size represents the absolute value of the difference between two hemispheres in mean node efficiency (right-left).

The hemispheric network nodes exhibiting significant between-hemisphere differences in nodal efficiency (FDR-corrected *P* < 0.05) in each group are shown in **Figure 4**. Only four nodes (512 in total) exhibited rightward asymmetry, and no node exhibited leftward asymmetry in the AD group. There were 42 nodes that exhibited rightward asymmetry and 43 nodes that exhibited left asymmetry in the MCI group. In the NC group, 5 nodes exhibited rightward asymmetry, and three nodes exhibited left asymmetry. For the MCI group, the regions/nodes with a rightward asymmetry in nodal efficiency were mostly located in the inferior frontal gyrus, precentral gyrus, superior parietal gyrus, paracentral lobule, postcentral gyrus, precuneus, CUN, and supramarginal gyrus, lingual gyrus, superior temporal gyrus, superior temporal gyrus, middle temporal gyrus, inferior temporal gyrus, fusiform gyrus, parahippocampal gyrus, lenticular nucleus, and insula. In the AD and NC groups, the regions exhibiting rightward asymmetry were located in a subset of the above-mentioned areas.

The leftward asymmetric nodes for the MCI group were mainly located in the anterior cingulate and paracingulate gyri, orbital part of the superior frontal gyrus, medial orbital part of the superior frontal gyrus, orbital part of the inferior frontal gyrus, orbital part of the middle frontal gyrus, triangular part of the inferior frontal gyrus, olfactory cortex, supplementary motor area, median cingulate, paracingulate gyri, precuneus, posterior cingulate gyrus, calcarine fissure, and surrounding cortex, superior temporal gyrus, superior temporal gyrus of the temporal pole, middle temporal gyrus, HIP, parahippocampal gyrus, caudate, thalamus, and lenticular nucleus. These regions mostly covered leftward asymmetric regions in the NC group, except for the posterior cingulate gyrus.

### Between-Group Differences in the Asymmetry of Nodal Efficiency

**FIGURE 5:**
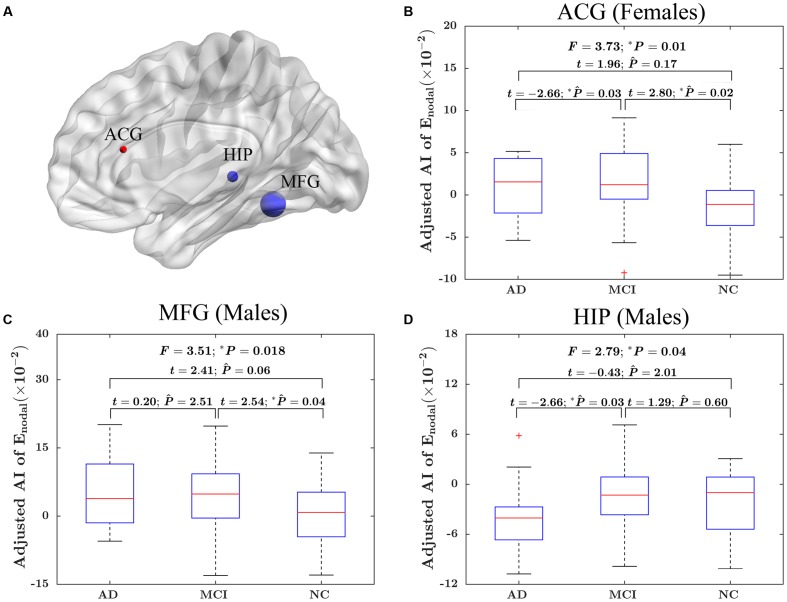
The “sex × group” interaction effects on the AI of nodal efficiency. **(A)** Three-dimensional representations of the significant “sex × group” interaction effects on AI of nodal efficiency (uncorrected *P* < 0.05), The nodes in blue represent the significant difference in males, and the node in red represents significant difference in females. The node size represents the F value when comparing the AI of nodal efficiency among the three groups. **(B)** The box plots of the “sex × group” interaction effects on the AI of nodal efficiency in ACG in females. **(C)** The box plots of the “sex × group” interaction effects on the AI of nodal efficiency in MFG in males. **(D)** The box plots of the “sex × group” interaction effects on the AI of nodal efficiency in HIP in males. Before conducting the group comparisons, the data were adjusted for whole-brain ICV, hemispheric ICV difference, age, and TR and TE from diffusion-weighted imaging. $\overset{\;\wedge }{P}$ represents the Bonferroni-corrected *P*-value of *post hoc* analysis.

At the nodal efficiency level, there was significant “sex × group” interaction effect on three nodes (uncorrected *P* < 0.05). These nodes were mainly located around MFG, HIP and ACG. More specifically, the MFG and HIP showed significant group difference in the AI of nodal efficiency in male only (MFG: *F* = 3.51; *P* = 0.018; HIP: *F* = 2.79, *P* = 0.04), whereas ACG was significant group difference only in female (*F* = 3.73; *P* = 0.01). More specifically, for females, there were significantly difference in the asymmetry of nodal efficiency in ACG between AD group and MCI group (*t* = –2.66; *P*_Bonferroni_ = 0.03) and between MCI group and NC group (*t* = 2.80; *P*_Bonferroni_ = 0.02). For males, there were significantly difference in the asymmetry of nodal efficiency in MFG between MCI group and NC group (*t* = –2.54; *P*_Bonferroni_ = 0.04) and in HIP between AD group and MCI group (*t* = *–*2.66; *P*_Bonferroni_ = 0.03). The detailed results were shown in **Figure 5**.

**FIGURE 6:**
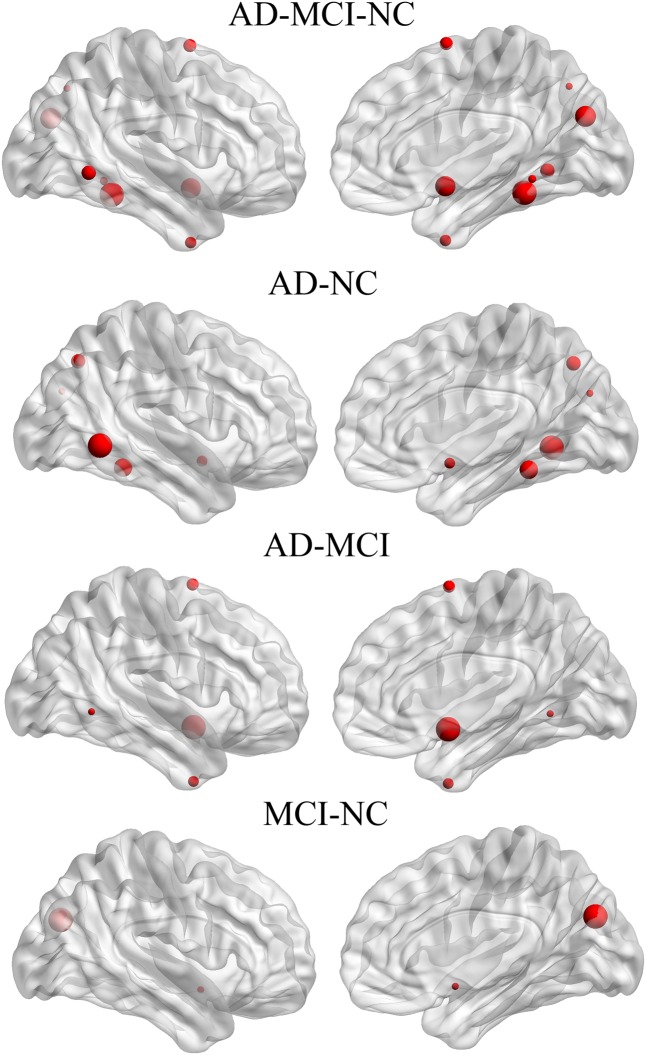
Three-dimensional representations of the nodes with significant between-group differences in the AI of nodal efficiency. The node size represents the *F*-value of GLM when comparing the AI of nodal efficiency among the three groups, and represents the *t*-value of *post hoc* analysis (no FDR).

**FIGURE 7:**
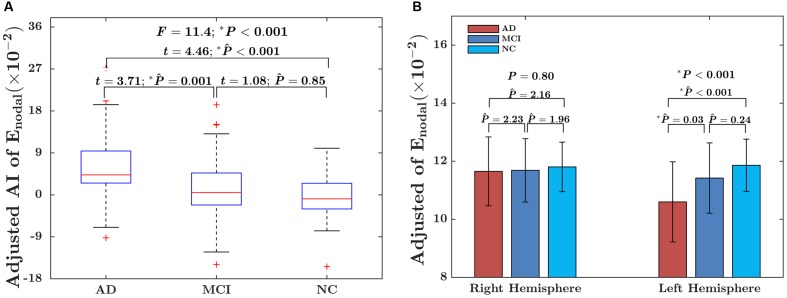
**(A)** Box plot of group differences in the AI, based on the mean AI of observed significant nodes. The data were adjusted for age, gender, whole-brain ICV, hemispheric ICV differences, and TR and TE from diffusion-weighted imaging. **(B)** Bar charts depicting group differences in the mean nodal efficiency in each hemisphere. The data were adjusted for age, sex, hemispheric ICV, and TR and TE from diffusion-weighted imaging. *P* represents the *P*-value of GLM, and $\overset{\;\wedge }{P}$ represents the Bonferroni-corrected *P*-value of *post hoc* analysis.

The significant group difference in the AI of nodal efficiency is illustrated in **Figure 6**. Among these three groups, we found that eight nodes (uncorrected *P* < 0.05; 512 in total) showed a significant group effect on the AI of nodal efficiency. These nodes were primarily located in the anterior and posterior portion of the inferior temporal gyrus, lingual gyrus, CUN, and precuneus, AFG, supplementary motor area, and anterior parahippocampal gyrus (**Table 2**). *Post hoc* analysis showed that most of the five disease-related nodes exhibited increased rightward asymmetry in the AD group compared with the NC group, except for a small portion of the anterior inferior temporal gyrus and supplementary motor area. There were 4 nodes exhibiting increased rightward asymmetry in patients with AD compared to those with MCI, mainly located in a small portion of the anterior inferior temporal gyrus, AFG, anterior parahippocampal gyrus and supplementary motor area. Two nodes, including the CUN and anterior parahippocampal gyrus, showed significant group differences between the MCI and NC groups, with a higher AI of nodal efficiency observed in patients with MCI compared to NC. Similar to the network efficiency, there was a significant group effect on the mean AI of nodal efficiency and the mean nodal efficiencies of the left hemisphere based on the regions affected by disease (**Figure 7**).

**Table 2:** The percentage of voxels in AAL regions that exhibited a significant group difference in nodal efficiency asymmetry among three groups.

AAL regions	Percentage	Type
Inferior temporal gyrus	21.17	Association
CUN	17.00	Association
Supplementary motor area	16.88	Association
AFG	15.81	Association
Superior occipital gyrus	8.44	Association
Lingual gyrus	5.23	Association
Olfactory cortex	4.40	Limbic
Anterior parahippocampal gyrus	2.50	Paralimbic
Angular gyrus	1.78	Association
Precuneus	1.55	Association
Amygdala	1.31	Subcortical
Calcarine fissure and surrounding cortex	0.95	Primary
Superior parietal gyrus	0.95	Association
Lenticular nucleus, putamen	0.95	Subcortical
Caudate nucleus	0.59	Subcortical
Gyrus rectus	0.48	Paralimbic


#### Relationship between AI and RAVLT

**FIGURE 8:**
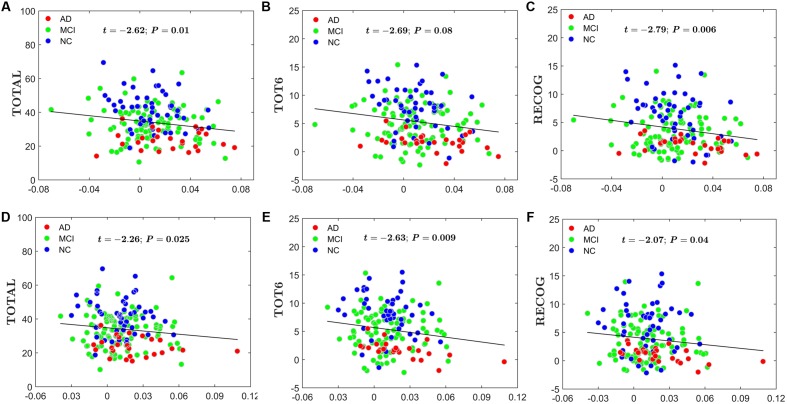
Scatter plots of the AI of the global network and local network efficiency and RAVLT-related scores. The red circles represent AD patients; the green circles represent MCI patients; the blue circles represent NC subjects. **(A)** The significant correlations between the AI of the global network efficiency and TOTAL. **(B)** The significant correlations between the AI of the global network efficiency and TOT6. **(C)** The significant correlations between the AI of the global network efficiency and RECOG. **(D)** The significant correlations between the AI of the local network efficiency and TOTAL. **(E)** The significant correlations between the AI of the local network efficiency and TOT6. **(F)** The significant correlations between the AI of the local network efficiency and RECOG.

**FIGURE 9:**
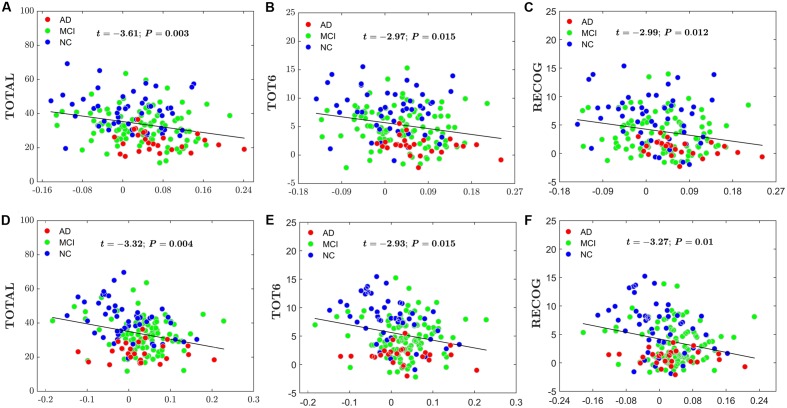
Scatter plots of the AI of the nodal efficiency and RAVLT-related scores. The red circles represent AD patients; the green circles represent MCI patients; the blue circles represent NC subjects. **(A)** The significant correlations between the AI of the nodal efficiency and TOTAL in AFG. **(B)** The significant correlations between the AI of the nodal efficiency and TOT6 in AFG. **(C)** The significant correlations between the AI of the nodal efficiency and RECOG in AFG. **(D)** The significant correlations between the AI of the nodal efficiency and TOTAL in CUN. **(E)** The significant correlations between the AI of the nodal efficiency and TOT6 in CUN. **(F)** The significant correlations between the AI of the nodal efficiency and RECOG in CUN.

For the global network efficiency and local network efficiency, there were significant negatively correlations between the AI and any RAVLT scores (For AI of *E*_global_, TOTAL: *t* = –2.62, *P* = 0.01; TOT6: *t* = –2.69, *P* = 0.08; RECOG: t = –2.79, *P* = 0.006. **Figures 8A–C**. For AI of *E*_local,_ TOTAL: *t* = –2.26, *P* = 0.0025; TOT6: *t* = –2.63, *P* = 0.009; RECOG: *t* = –2.07, *P* = 0.04; **Figures 8D–F**). At the nodal efficiency level, 2 nodes (FDR-corrected *p* < 0.05), mainly located around AFG and CUN, exhibited significant negative correlations with RAVLT scores. (For AFG, TOTAL: *t* = –3.61, *P* = 0.003; TOT6: *t* = –2.97, *P* = 0.015; RECOG: *t* = –2.99, *P* = 0.012; **Figures 9A–C**. For CUN, TOTAL: *t* = –3.32, *P* = 0.004; TOT6: *t* = –2.93, *P* = 0.015; RECOG: *t* = –3.27, *P* = 0.010; **Figures 9D–F**).

## Discussion

In the present study, we investigated alterations in the topological asymmetry between the hemispheric brain WM networks of AD patients and MCI patients. Our main findings are as follows: (1) the hemispheric brain WM networks showed an aberrant rightward topological asymmetry in AD patients, but not in the early phase of the disease (MCI); (2) there were significant group differences in the topological properties only in the left hemisphere, suggesting a key role of the left hemisphere in the aberrant topological asymmetry in AD; and (3) the degree of rightward asymmetry in nodal efficiency was increased primarily around the temporal lobe in patients with AD compared with NC subjects. (4) the aberrations of hemispheric network topological asymmetry of AD and MCI patients were correlated with the memory performance. These findings provide direct evidence of changes in network asymmetry in AD compared to normal aging and extend our understanding of the neurophysiologic mechanisms of AD from a network asymmetry perspective.

### Disrupted Hemispheric Brain WM Network in AD and MCI

Previous neuroimaging studies have reported reduced network efficiency of the whole-brain WM network in AD patients, compared with NC subjects (Lo et al., 2010; Bai et al., 2012; Reijmer et al., 2013). Interestingly, we found similar results at the hemispheric brain WM network level. That is, compared with NC subjects, the network efficiency of patients with AD was significantly reduced in the left hemisphere. Consistent with the present findings, a disrupted hemispheric brain WM network has been previously reported by comparing 15 AD patients and NC subjects (Daianu et al., 2013). Taken together, these results consistently support the longstanding notion that AD is a syndrome of disconnection (Xie and He, 2012; Tijms et al., 2013; Dai and He, 2014). Notably, compared with either NC subjects or AD patients, the network efficiency of MCI patients showed no significant reduction in network efficiency of either hemisphere. This finding is consistent with the concept of MCI as a transitional stage between NC and AD (Petersen et al., 1985), and a trend for AD < MCI < NC was observed when comparing the hemispheric network efficiency among the three groups in both the left hemisphere and right hemisphere.

#### Within-Group Asymmetry

In addition, we found that NC subjects exhibited no significant difference in two hemispheric brain WM networks, suggesting that the two hemispheres showed similar intra-connected pathways in NC. However, conflicting results have been reported. For example, Caeyenberghs and Leemans (2014) reported leftward asymmetry in network efficacies in NC. This inconsistency is likely due to the differences in network resolution, weighting strategy for network edges, age range of samples. The network construction methods can dramatically affect WM network topological properties (Zalesky et al., 2010; Bassett et al., 2011; Zhong et al., 2015), and the construction methods differed between the previous study (Caeyenberghs and Leemans, 2014) and the present study: Caeyenberghs and Leemans (2014) involved 45 nodes (low resolution), whereas we used 512 nodes in the present study (high resolution). In addition, the number of fibers was defined as edge weights by Caeyenberghs, while we used the FA value as weight.

Notably, the AD patients exhibited significant right-greater-than-left asymmetry of network efficiency, suggesting that the left hemisphere is intra-connected in a more poorly integrated way with less efficient communication at the hemispheric level in AD. While there are no comparable structural data involving hemispheric network efficiency in patients with AD, our findings are consistent with earlier studies showing that the left hemisphere lost GM faster than the right in AD patients (Thompson et al., 2003, 2007).

### Atypical Topological Asymmetry in AD and MCI Patients

Compared with the NC subjects, the AD patients exhibited significantly increased rightward asymmetry in both local network efficiency and global network efficiency. Considering the symmetrical topological organization in the normal aging brain, this aberrant hemispheric asymmetry in AD is likely related to a mismatch in disease-related changes in structural properties within the two hemispheres. Compatibly, Thompson et al. (Thompson et al., 2007) demonstrated that cortical atrophy occurred earlier and progressed faster in the left hemisphere than in the right in AD, indicating that the left hemisphere was more severely impaired than the right and that there was a faster left hemisphere degeneration in AD. Interestingly, our data reveled a disease-related disruption in local network efficiency in the left hemisphere but a slight change in the right hemisphere. Biochemically, the significant decrease in network efficiency may be related to the β-amyloid-related protein in AD (Tsai et al., 2009).

At the nodal level, two nodes (the parahippocampal gyrus and CUN) showed abnormal topological asymmetry in both AD and MCI patients compared with NC subjects. This atypical topological asymmetry in the parahippocampal gyrus may be associated with structural asymmetry. For example, Kim et al. (Kim et al., 2012) studied the cortical thickness of MCI patients, mild AD patients and moderate-to-severe AD patients and found a progressive reduction in cortical asymmetry in the parahippocampal gyrus. Liu et al. reported a reduced FA value in right parahippocampal WM in both AD and MCI patients (Liu et al., 2011). For CUN, the abnormalities in functional regional homogeneity have been also reported in AD (Wang et al., 2006; He et al., 2007). Given the important role of the parahippocampal gyrus (Staresina et al., 2011) and CUN (Addis et al., 2004) in episodic memory, it is possible that the observed atypical topological asymmetry in these regions may be related to impaired memory of AD and MCI patients.

Compared with the NC subjects, several regions were identified to have abnormal asymmetry in nodal efficiency only in AD, including the precuneus, temporal regions, and lingual gyrus. Given the important role of the precuneus in communication (Hagmann et al., 2007; Gong et al., 2009), which is a hub region in both structure network an resting-state network, our findings provide further support to the notion that these brain hubs may be preferentially affected in AD (Buckner et al., 2009; Jacobs et al., 2013; Dai et al., 2015). The disruptions of the hub regions may indicate more isolated network architecture in AD, presumably leading to the observed deficits in overall global integration. GM loss in precuneus of the AD brain has also been consistently found by applying voxel-based morphometry (VBM) (Derflinger et al., 2011). For AFG, abnormalities in this region of AD patients have also been reported in previous studies. He et al (He et al., 2007) have reported that the fusiform gyrus showed increased blood oxygenation level-dependent fluctuations (LFBF) in AD patients. Yao et al (Yao et al., 2010) have found the abnormalities of interregional correlation in the fusiform gyrus in AD patients. For lingual gyrus, Sun et al (Sun et al., 2014) have found abnormal nodal efficiency in the lingual gyrus in AD patients. For inferior temporal gyrus, several studies have confirmed that the temporal gyrus atrophy in AD patients was correlated with changes of white matter (i.e., microstructure, number of synapses) (Scheff et al., 2011; Huang et al., 2012; Delli Pizzi et al., 2015). In addition, biochemically, Johnson et al evaluated ^18^F T807, a PET radiopharmaceutical selective for tau pathology by comparing normal individuals to MCI or mild AD dementia patients. They found that ^18^F T807 binding in the MCI/AD patients was especially high in inferior temporal lobe (Johnson et al., 2016). The results of these studies give us some reasonable explanations for the observed abnormal asymmetry in these regions from different perspectives.

The increased degree of rightward asymmetry in nodal efficiency is primarily due to changes in the left hemisphere, which has been well-documented to dominantly process language (Gotts et al., 2013). Therefore, our results are to some extent consistent with the clinical symptoms of AD, and imply that atypical topological asymmetries may relate to the impairment in verbal memory, language and other cognitive functions in AD. Notably, there were more nodes exhibiting significant asymmetry in AD patients than those in MCI patients, compared with NC subjects, further supporting the transitional role of the MCI between the NC and AD.

### Relationship between Topological Asymmetry and the Memory Performances

We examined the correlation between the AI of network metrics and RAVLT scores (i.e., TOTALL, TOT6 and RECOG). We found that subjects with higher level rightward asymmetry in local network efficiency and global network efficiency had lower verbal memory performance. Also, subjects with higher level rightward asymmetry in nodal efficiency of two cortical regions (i.e., AFG and CUN) had lower verbal memory performance. Several previous research results were consistent with the present findings. For example, Reijmer et al. (2013) found that memory performance (including RAVLT scores) was correlated with local efficiency and global efficiency in WM network in AD. At nodal level, He and his colleagues found (He et al., 2007) that MMSE score in the AD patients group were significant positive correlations with ReHo in CUN. In addition, previous morphological studies also have demonstrated the AD-related structural changes within the brain is closely related to RAVLT measures (Estévez-González et al., 2003; Balthazar et al., 2010; Stonnington et al., 2010; Moradi et al., 2016). Notably, Derflinger et al. (2011) found that the performance of language-based neuropsychological tests was correlated with lateralization of GM loss to the left hemisphere in AD. This agreement further validates our finding that the rightward asymmetry was mainly contributed by the changes of the topological properties of AD patients in the left hemisphere. Thus our results of aberrant hemispheric asymmetry likely reflect the breakdown of function connections that influences memory performances of the AD patients. The present findings support a general trend in the literature showing a direct correspondence between clinical symptomatology and the underlying anatomical structures supporting network efficiency and suggest the usefulness of brain network properties as potential biomarkers for diagnosis and evaluation of the severity of the disease as well as understanding the pathophysiologic mechanisms.

#### Limitations and Future Works

Finally, a few limitation should be addressed. First, a tensor-model-based deterministic tractography method was utilized to reconstruct the structural brain networks in this study. It is has been well-known that this fiber tracking method cannot handle the “fiber crossing” problem (Mori and van Zijl, 2002). Topological asymmetry analysis based on other methods such as probabilistic tractography or other imaging models such as diffusion spectrum MRI or high-angular-resolution diffusion imaging (HARDI) can be used in further studies. Second, handedness has been shown to impact the network efficiency asymmetries of human brains (Li et al., 2014). However, the present study did not include handness information; therefore, no assessment of the effect of handedness on network asymmetries was made. Next, the current study did not analyze the relationship between changes in network asymmetry and cortical/subcortical atrophy. Given the WM alteration could be related to cortical/subcortical atrophy (Huang et al., 2012; Delli Pizzi et al., 2015), we will evaluate changes of asymmetry- cortical/subcortical atrophy relationships in AD the future. It is highly desired to explicitly evaluate changes of asymmetry-cognition relationships in AD the future. At the end, the sample size of the present study is relatively small (48 NC subjects, 95 MCI and 25 AD patients). Give the ADNI2 subjects are continuously being updated, future work is encouraged to assess a larger sample size to verify our current findings.

## Conclusion

The present study revealed increased rightward asymmetry in hemispheric brain WM networks from NC to MCI to AD, and the rightward asymmetry is attributed to aberration of topological properties in the left hemisphere in patients. In addition, the degree of the rightward asymmetry of topological properties was also correlated with memory performance. These findings provide another angel to understand the pathological mechanisms in AD and highlight the potential for brain network asymmetry-based biomarkers for AD.

## Author Contributions

CY and SZ designed experiments; CY and LW collected and sorted data; XZ and CY carried out experiments, data analysis and statistical analysis; LJW and LW assistance for statistical analysis; CY, SZ, and XZ wrote the manuscript; SZ, LJW, and SN revised the manuscript.

## Conflict of Interest Statement

The authors declare that the research was conducted in the absence of any commercial or financial relationships that could be construed as a potential conflict of interest.

## Footnotes

1 http://www.adni-info.org/

2 http://www.nitrc.org/projects/gretna/

3 http://www.fil.ion.ucl.ac.uk/spm/software/spm8/

## Abbreviations

ACG: anterior cingulate gyrus
AD: Alzheimer’s disease
ADNI: Alzheimer’s Disease Neuroimaging Initiative
AFG: anterior fusiform gyrus
AI: asymmetry index
CDR: Clinical Dementia Rating
CUN: cuneus
FA: fractional anisotropy
FDR: false discovery rate
GLM: general linear models
GM: gray matter
HIP: hippocampus
ICV: intracranial volume
MCI: mild cognitive impairment
MFG: middle part of fusiform gyrus
MMSE: Mini Mental Status examination
MNI: Montreal Neurological Institute
NC: normal control
RAVLT: Rey’s Auditory Verbal Learning Test
RECOG: 30-min delay recall scores
ROI: region of interest
TE: echo time
TOT6: scores of trail 6
TOTAL: total score of the first 5 learning trials
TR: repetition time
WM: white matter
